# Application of quantitative T1, T2 and T2* mapping magnetic resonance imaging in cartilage degeneration of the shoulder joint

**DOI:** 10.1038/s41598-023-31644-2

**Published:** 2023-03-20

**Authors:** Guijuan Cao, Shubo Gao, Bin Xiong

**Affiliations:** 1 Department of Radiology, Union Hospital, Tongji Medical College, Huazhong University of Science and Technology, Jiefang Avenue #1277, 430022 Wuhan, Hubei China; 2 Department of Radiology, the Central Hospital of Wuhan, Tongji Medical College, Huazhong University of Science and Technology, Wuhan, China; 3 Department of Interventional Radiology, The First Affiliated Hospital of Guangzhou Medical University, The First Affiliated Hospital of Guangzhou Medical University, Guangzhou, China

**Keywords:** Diseases, Medical research

## Abstract

To investigate and compare the values of 3.0 T MRI T1, T2 and T2* mapping quantification techniques in evaluating cartilage degeneration of the shoulder joint. This study included 123 shoulder joints of 119 patients, which were scanned in 3.0 T MRI with axial Fat Suppression Proton Density Weighted Image (FS-PDWI), sagittal fat suppression T2 Weighted Image (FS-T2WI), coronal T1Weighted Image (T1WI), FS-PDWI, cartilage-specific T1, T2 and T2* mapping sequences. Basing on MRI images, the shoulder cartilage was classified into grades 0 1, 2, 3 and 4 according to the International Cartilage Regeneration & Joint Preservation Society (ICRS). The grading of shoulder cartilage was based on MRI images with ICRS as reference, and did not involve arthroscopy or histology.The T1, T2 and T2* relaxation values in the superior, middle and inferior bands of shoulder articular cartilage were measured at all grades, and the differences in various indicators between groups were analyzed and compared using a single-factor ANOVA test. The correlation between T1, T2 and T2* relaxation values and MRI-based grading was analyzed by SPSS software. There were 46 shoulder joints with MRI-based grade 0 in healthy control group (n = 46), while 49 and 28 shoulder joints with grade 1–2 (mild degeneration subgroup) and grade 3–4 (severe degeneration subgroup) in patient group (n = 73), accounting for 63.6% and 36.4%, respectively. The T1, T2 and T2* relaxation values of the superior, middle and inferior bands of shoulder articular cartilage were significantly and positively correlated with the MRI-based grading (*P* < 0.01). MRI-basedgrading of shoulder cartilage was markedly associated with age (r = 0.766, *P* < 0.01). With the aggravation of cartilage degeneration, T1, T2 and T2* relaxation values showed an upward trend (all *P* < 0.01), and T1, T2 and T2* mapping could distinguish cartilage degeneration at all levels (all *P* < 0.01). The T1, T2 and T2* relaxation values were significantly different between normal group and mild degeneration subgroup, normal group and severe degeneration subgroup, mild degeneration subgroup and severe degeneration subgroup (all *P* < 0.05). Quantitative T1, T2 and T2* mapping can quantify the degree of shoulder cartilage degeneration. All these MRI mapping quantification techniques can be used as critical supplementary sequences to assess shoulder cartilage degeneration, among which T2 mapping has the highest value.

Osteoarthritis (OA) is a degenerative joint disease characterized by damage to articular cartilage. The main histological manifestations are progressive collagen fibrous disorganization of the articular cartilage, free water content increased, and depletion of glycosaminoglycans (GAG). Multiple stages and grades of degeneration can co-occur^1–5^. Early articular cartilage degeneration is related to the change the composition and distribution of collagen fibers, mucin and water molecules in articular cartilage^6^. Arthroscopic assessment can be considered to be the gold standard for OA diagnosis^7,8^. But arthroscopy is an invasive examination with high cost, low acceptance degree of patients, and can not be dynamically detected, so it has no screening and disease evaluation value.Conventional magnetic resonance imaging (MRI) image can evaluate the morphological changes in articular cartilage; however, it remains difficult to identify the subtle changes in articular cartilage matrix on the MRI images. In particular, the biochemical components and structure of the articular cartilage in patients with early osteoarthritis have changed before the onset of obvious clinical symptoms. The repair ability of articular cartilage is limited, and it does not regenerate if it is seriously damaged. Therefore, it is of great significance to find reliable and effective auxiliary examination methods in clinic for the diagnosis and treatment of OA. MRI mapping quantification techniques is important for the early detection of articular cartilage degeneration, which can support clinical diagnosis and treatment decisions^9,10^.

Articular cartilage is hyaline cartilage composed of chondrocytes and an extracellular matrix consisting mainly of water, highly organized collagen polysaccharides, and collagen glycosaminoglycans^11^. T2 mapping is sensitive to the changes in the water content, collagen fiber content, and orientation of the cartilage^12^. T2 mapping and T2* mapping were related to water content and the interaction between water molecules and collagen fibers. T2* mapping differs from T2 mapping in that the former uses a magnetic gradient rather than 180° polyphasic RF pulses, reflecting the inhomogeneity of the magnetic field^13^. T2* mapping is more sensitive to the structural changes of cartilage rather than the water content due to its shorter echo time (TE) compared to T2 mapping^14^. The T2* mapping image may be more sensitive to the collagen structure of the cartilage rather than the water content. Previous literature also points out that T2 mapping and T2* mapping images are susceptible to magnetic sensitivity artifacts (e.g., magic angle effect), which leads to a decline in diagnostic efficiency^15,16^. T1 mapping is relatively new MRI that can reflect the water content in cartilage at the early stage of cartilage injury^17–19^. Thus, the main components affecting T1, T2 and T2* values are different, and the sensitivity and specificity of the three sequences to cartilage degeneration are also different.

In recent years, quantitative techniques, such as T1, T2 and T2* mapping, have been applied to evaluate the cartilage degeneration of the knee joint, hip joint, and ankle joint^20–25^, but there are only a few reports on shoulder cartilage^26–28^. Typically, only one or two quantitative techniques are applied on shoulder cartilage, and the simultaneous application of T1, T2 and T2* mapping in shoulder cartilage has not been reported. Human shoulder cartilage is closely attached to the bone surface of shoulder joint, and the articular cartilage of shoulder joint has a curved shape^11^. The morphology of shoulder cartilage in different populations also has specific differences. However, due to the thickness of MRI image layer and the existence of partial volume effect, diagnostic physicians cannot recognize some subtle changes with the naked eye, and small lesions are easily missed. T1, T2 and T2* mapping quantitative technology, as a non-invasive imaging examination, can be used to detect the early degeneration of articular cartilage, as well as to quantitatively analyze the biochemical components and structures of articular cartilage^29–31^. Compared with the MRI sequences, the quantitative T1, T2 and T2* mapping sequences can better evaluate the changes in the composition and structure of articular cartilage and is more sensitive to the detection of articular cartilage damage and degeneration^32^.

In this study, the T1, T2 and T2* relaxation values of shoulder cartilage in 3-TESLA MRI were compared between the normal and patient groups to investigate and compare the values of 3.0 T MRI T1, T2 and T2* mapping quantification techniques in evaluating cartilage degeneration of the shoulder joint.

## Material and methods

This study was approved by the institutional review board of the Central Hospital of Wuhan, and the study procedures were performed in accordance with the Declaration of Helsinki’s ethical principles for medical research involving human subjects. Written informed consent was waived by Ethics Committee of Wuhan Central Hospital.

### Study population

The study group consisted of 119 patients (four of whom underwent MRI scans of bilateral joints) due to shoulder discomfort from November 2020 to November 2021. In the patient group, a total of 77 shoulder joints were detected, including 27 male shoulder joints and 50 female shoulder joints, the patients ranged in age from 33 to 83 years old with an average age of 55.1 ± 12.8 years old. There were 46 healthy controls in the normal group, including 21 male shoulder joints and 25female shoulder joints, the healthy group ranged in age from 18 to 61 years, with an average age of 25.6 ± 8.0 years. Inclusion criteria: no shoulder trauma or surgical history; no congenital dysplasia of shoulder joint; BMI was within the normal range of 18.5–24 kg/m^2^, so the influence of height and weight on the measurement of the three mapping values was excluded. Exclusion criteria: patients with contraindication and claustrophobia who did not complete an MRI scanning of the shoulder joint; patients with poor image quality that affected evaluation; asymptomatic patients who were engaged in heavy manual labor and professional athletes and had the possibility of shoulder injury.

### MRI methods

The 3.0 T MR imaging (MAGNETOM SKYRA) was conducted using a special coil for shoulder joint scanning, which wraps around the surface of the shoulder joint. The sandbags were used to pad the distal end of the shoulder joint to make it as high level as possible with the proximal end to improve the patient's comfort. MRI scanning was performed after fixing the distal end of the shoulder joint with the sandbags. During MRI scanning, the shoulder joint was placed at the center of the field of view, and its long axis should be consistent with the direction of the main magnetic field as far as possible. The MRI protocol included axial Fat Suppression Proton Density Weighted Image (FS-PDWI), sagittal fat suppression T2 Weighted Image (FS-T2WI), coronal T1Weighted Image (T1WI), FS-PDWI, T1, T2 and T2* mapping sequences .Of note, the sequence setting in this study favoured shoulder cartilage image quality ,which necessitate various pulse sequences with T1-, T2- and PD-weighting in coronal, sagittal and axial planes. In order to ensure the accurate positioning of the central level on shoulder cartilage image, the coronal T1, T2 and T2* mapping scanning were performed based on the coronal FS-PDWI scanning.

Further details on scanning parameters are provided in Table 1.

**Table 1:** MRI scan sequences and parameters of the shoulder joint.

Sequence parameter	PD-qtse-fs-tra	t2-qtse-fs-sag	t1-qtse-cor	t2-tse-Dixon-cor	t1-mapping-cor	t2-mapping-cor	t2*-mapping-cor
TR (repetition time, ms)	3780	3500	600	3400	15	1000	445
TE (echo time, ms)	24	60	10	66	1.88	13.8,27.6,41.4,55.2,69	4.36,11.9,19.44,26.98,34.52
FOV (field of view, mm^2^)	160	160	160	160	160	160	160
In-plane resolution (mm)	0.6 × 0.6	0.6 × 0.6	0.6 × 0.6	0.6 × 0.6	0.6 × 0.6	0.6 × 0.6	0.6 × 0.6
Slice thickness (mm)	3	3	3	3	3	3	3
Slice gap (mm)	0.6	0.6	0.6	0.6	0.6	0.6	0.6
NEX (number of excitation)	1	1	1	1	1	1	1
FA (flip angle, °)	150	150	150	150	5/26	180	60
Bandwith (Hz/pixel)	217	217	250	320	280	230	260
TA (aquisition time, min)	1′59	1′15	1′20	2′1	1′43	4′36	2′10

### Image post-processing

**Figure 1:**
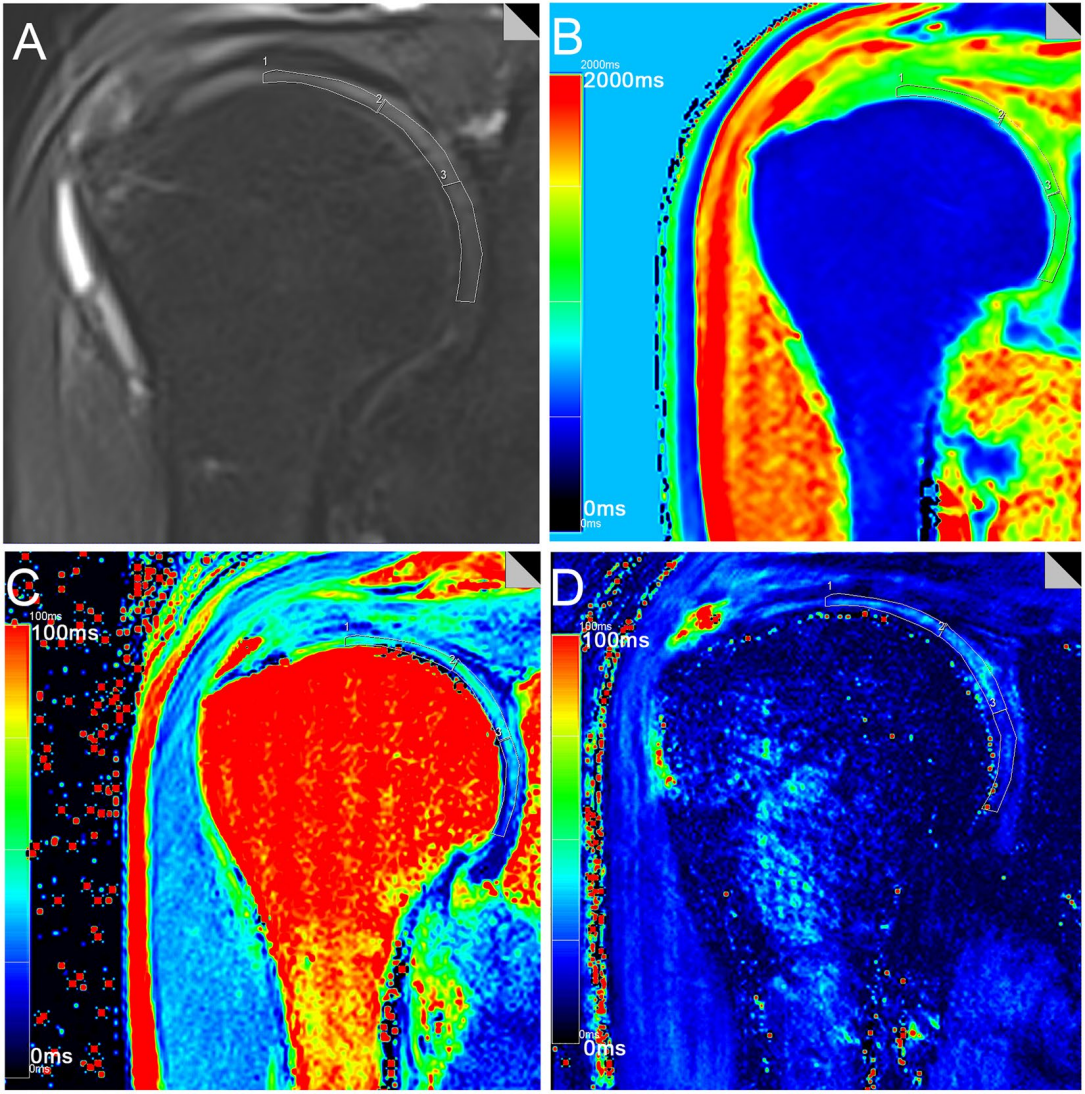
Referring to the position of cartilage on the coronal fat suppression T2WI image (**A**), T1, T2 and T2* relaxation values were assessed in three regions (superior, middle and inferior) from acromion end to glenoid by means of region of interest (ROI) analysis. f note, because of low cartilage thickness and a high degree of congruency between the articular surfaces, glenoidal and humeral cartilage layers were not reliably distinguishable. Therefore, ROI analysis included glenoidal and humeral cartilage as one combined entity. (**B**-**D**) are T1, T2 and T2* mapping pseudo-color maps and corresponding placement positions of ROI and T1, T2 and T2* mapping values, respectively.

**Figure 2:**
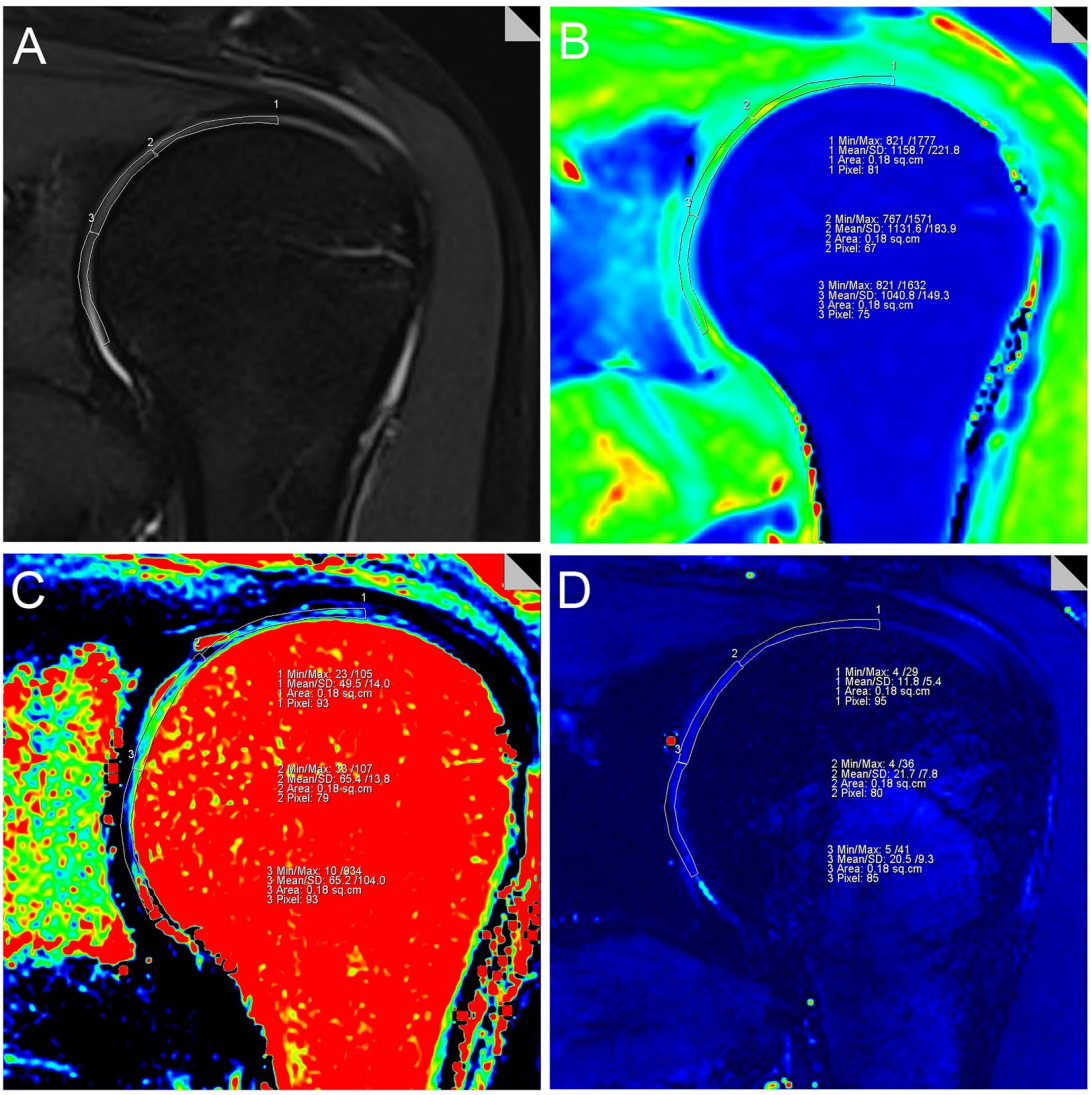
Male, 27 years old, grade 0 normal group, coronal adipose suppression T2WI sequence, T1, T2, T2* mapping pseudo-color and ROI T1, T2 mapping, T2* mapping values of left shoulder joint.

**Figure 3:**
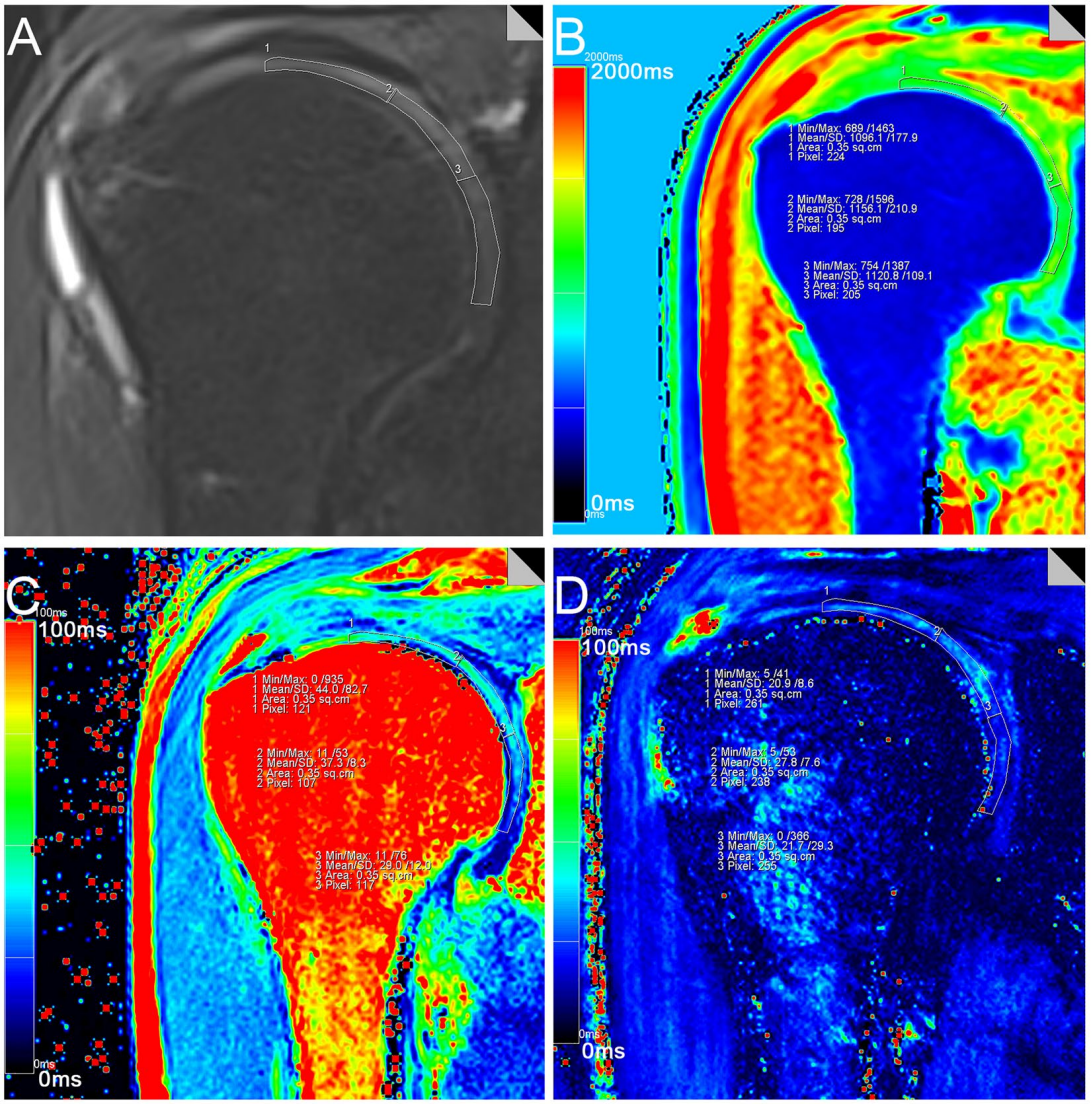
Female, 49 years old, grade 1 mild degeneration subgroup, coronal adipose suppression T2WI sequence, T1, T2, T2* mapping pseudo-color images and ROI T1, T2 mapping, T2* mapping values of right shoulder joint.

**Figure 4:**
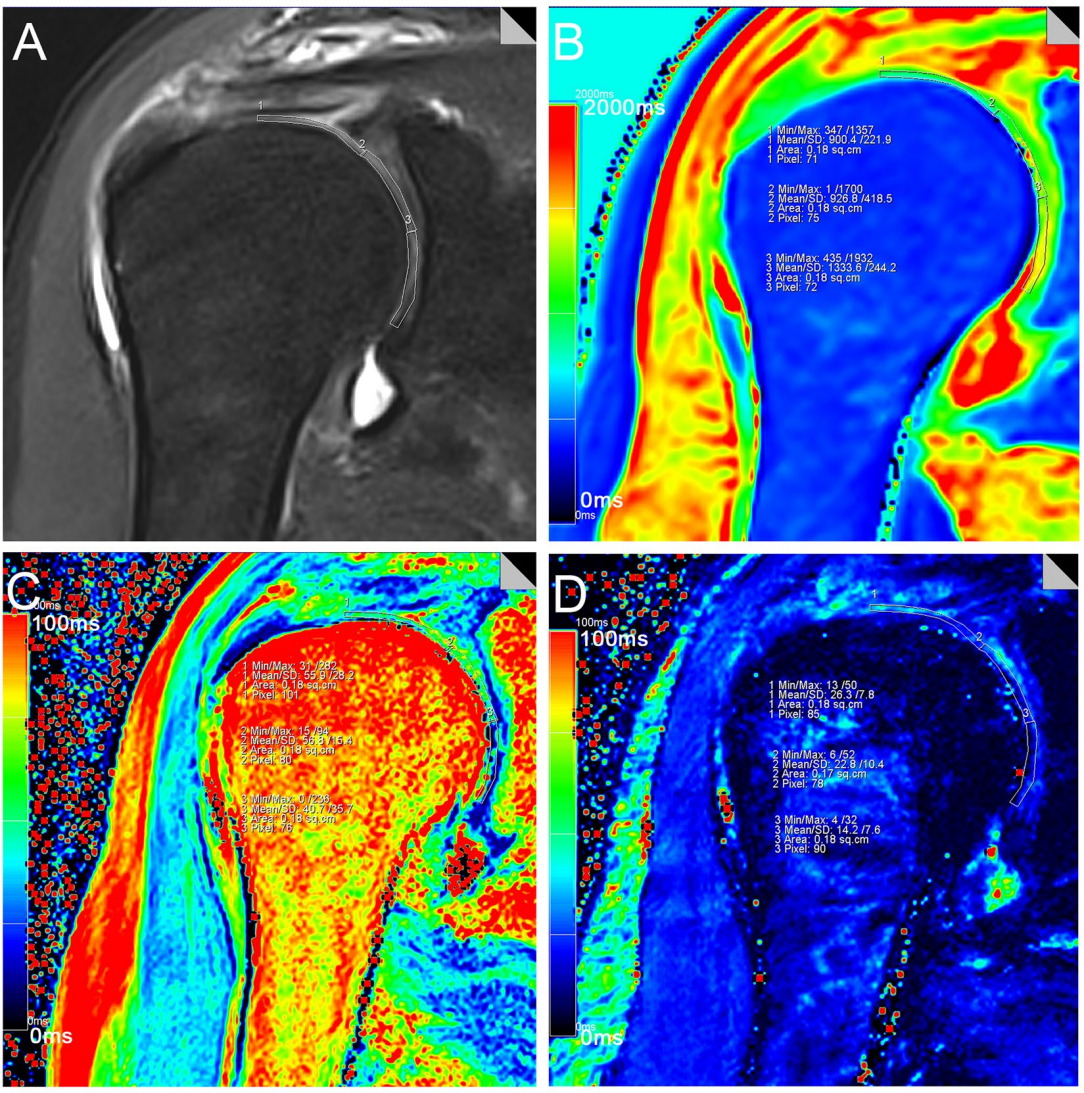
Female, 70 years old, grade 4 severe degeneration subgroup, coronal adipose suppression T2WI sequence, T1, T2, T2* mapping pseudo-color images and ROI T1, T2 mapping, T2* mapping values of right shoulder joint.

The T1, T2 and T2* relaxation values were calculated (MapIt, Siemens Healthcare) based on pseudo-color mapping, and then recorded using the MMWP workstation (Syngo Multimodality Workplace, Erlangen, Germany). The Siemens in vitro biochemical imaging-Maplt technology can detect the changes of macromolecules in the matrix in the cartilage at an early stage before the morphological changes. Due to the sensitivity of T1, T2, and T2* relaxation time to changes in collagen and water, the system automatically calculated and generated a mapping image, which could be used to quantitatively evaluate the changes of cartilage biochemical composition, and were widely used in various articular cartilage lesions.The window width and position were adjusted to make the shoulder cartilage display more clearly. After that, the Siemens processing workstation analysis software was used to directly draw the region of interest (ROI) on the pseudo-color images to measure T1, T2 and T2* relaxation values. Using the coronal FS-PDWI image as reference, the central layer with complete and clear display of shoulder cartilage was selected, and according to the previous literature^27,28^, the shoulder cartilage (glenohumeral cartilage) was divided into three equal parts by length from the acromial end to the glenoid pelvis, which were divided into superior band (weight-bearing zone), middle band (light weight-bearing zone) and inferior band (non-weight-bearing zone). Hence, the area of these three bands is the same (Fig. 1A). At the same time, the superior, middle and inferior bands of shoulder cartilage were measured at the same position of FS-PDWI, T1, T2 and T2* mapping coronal pseudo-color map to ensure the same area of measurement among the four groups. The results of FS-PDWI imaging and pseudo-color mapping are shown in Fig. 1A–D respectively. For reliability assessment ,each ROI was measured three times by two medical doctors and then averaged.Basing on MRI images, according to the standard ICRS grading (Supplemental Table 1)^33–35^, the shoulder cartilage was graded by at least two attending physicians in the Department of Radiology, which was divided into five grades: Grade 0, Grade 1, Grade 2, Grade 3, and Grade 4. Grade 0 was defined as the normal group (Fig. 2A–D), grade 1–2 as the mild degenerative group (Fig. 3A–D), and grade 3–4 as the severe degenerative group (Fig. 4A–D). If the results of the grading were inconsistent, the two physicians need to discuss together before making a decision.

### Statistical analysis

In this study, IBM SPSS software (Version 26.0; IBM Corp,Armonk, NY, USA) was used for statistical analyses. Students t test was used for continuous variables to compare between two groups and analysis of variance (ANOVA) test to compare among three groups.The T1, T2 and T2* relaxation values in the superior, middle and inferior bands of shoulder articular cartilage were in line with normal distribution, and were presented as 'x ± s. The differences in T1, T2 and T2* relaxation values between the superior, middle and inferior bands of shoulder cartilage in grade 0, grade 1, grade 2, grade 3 and grade 4 groups were compared by single factor ANOVA test. *P*-value of < 0.05 was considered statistically significant. The average value of T1, T2 and T2* relaxation values of superior, middle and inferior bands were obtained, and spearman’s correlation analysis was performed to determine the relationship between the average T1, T2 and T2* relaxation values and MRI-based grading, as well as that between age and MRI-based grading. Low correlation was considered when |r|< 0.5, and medium to high correlation was considered when |r|≥ 0.5. Two-by-two comparisons of T1, T2 and T2* relaxation values in the superior, middle and inferior bands of shoulder articular cartilage between normal, mild degeneration and severe degeneration groups were performed using the least significance difference (LSD) in single factor ANOVA test. *P*-value of < 0.05 was considered statistically significant.

## Results

### General results

**Figure 5:**
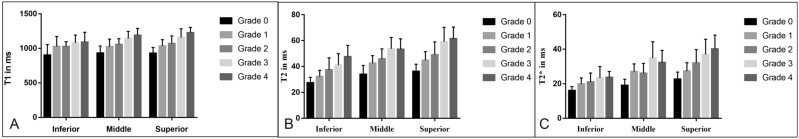
Bar diagram demonstrating T1, T2, and T2* relaxation times of the superior, middle and inferior band in various grades of cartilage degeneration. With the aggravation of cartilage degeneration, T1, T2, and T2* relaxation times of the superior, middle and inferior bands were all increased.

A total of 123 shoulder joint imaging images were included, and 46, 25, 24, 13 and 15 shoulder articular cartilages were detected in grade 0, grade 1, grade 2, grade 3 and grade 4 groups, respectively. Notably, 46 healthy controls had MRI-based grade 0, while 49 shoulder articular cartilages had grade 1–2 (mild degeneration subgroup) and 28 shoulder articular cartilages had grade 3–4 (severe degeneration subgroup), accounting for 63.6% and 36.4%, respectively. Single-factor ANOVA test analysis showed that with increasing cartilage degeneration, T1(Fig. 5A), T2(Fig. 5B) and T2* (Fig. 5C) values of the superior, middle and inferior band of shoulder cartilage in MRI-based grade 0–4 were statistically significant (all *P* < 0.01).

### Correlation between T1, T2, and T2* relaxation times and MRI-based grading of the superior, middle and inferior bands of shoulder cartilage

Spearman rank analysis showed that the T1 relaxation times of the superior, middle and inferior bands of shoulder cartilage were positively correlated with MRI-based grading (r = 0.711, 0.674 and 0.532, respectively, *P* < 0.01). Similarly, there was a highly positive correlation between T2 relaxation times and MRI-based grading (r = 0.773, 0.750 and 0.723, *P* < 0.01). There was a medium–high positive correlation between T2* relaxation times and MRI-based grading (r = 0.740, 0.725 and 0.636, *P* < 0.01). The correlation between T2 relaxation times and MRI-based grading was the highest, and the correlation between superior band and MRI-based grading was the highest in the three groups.

### Correlation between age and MRI-based grading, and correlation between age and average T1, T2 and T2* relaxation times in shoulder cartilage degeneration

**Figure 6:**
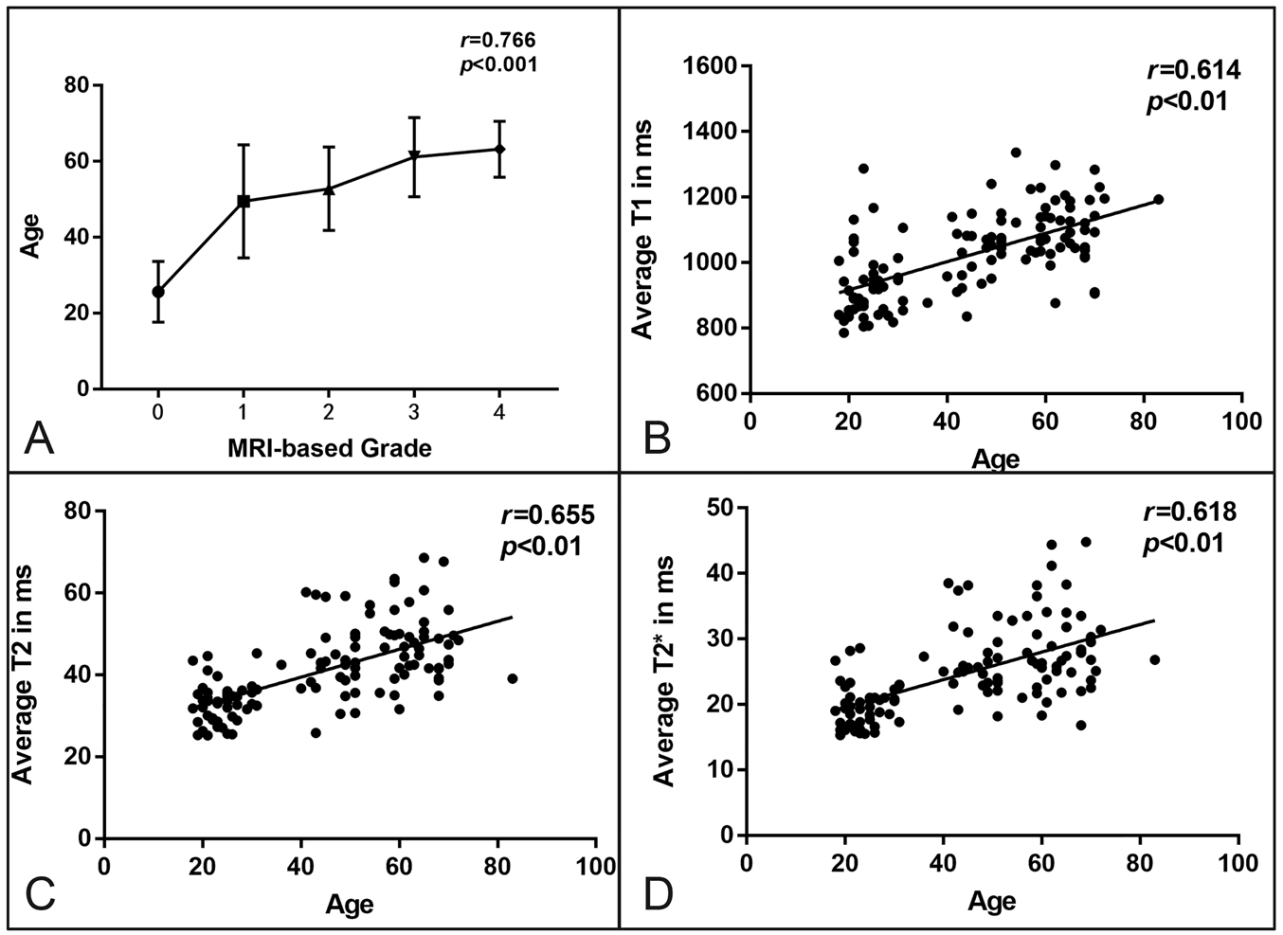
(**A**) Line chart illustrating age relative to the grades based on MRI image of cartilage degeneration. (**B-D**) Scatter plot illustrating average T1, T2 and T2* relaxation times relative to age.

The Spearman rank analysis revealed a correlation (correlation coefficient was 0.766, *P* < 0.01) between age and MRI-based grading(Fig. 6A). The average T1, T2 and T2* relaxation times of shoulder cartilage were all moderately positively correlated with age (r = 0.614, 0.655 and 0.618, respectively, all *P* < 0.01), (Fig. 6B–D).

### T1, T2 and T2* mapping to identify the degree of cartilage degeneration in the shoulder joint

**Figure 7:**
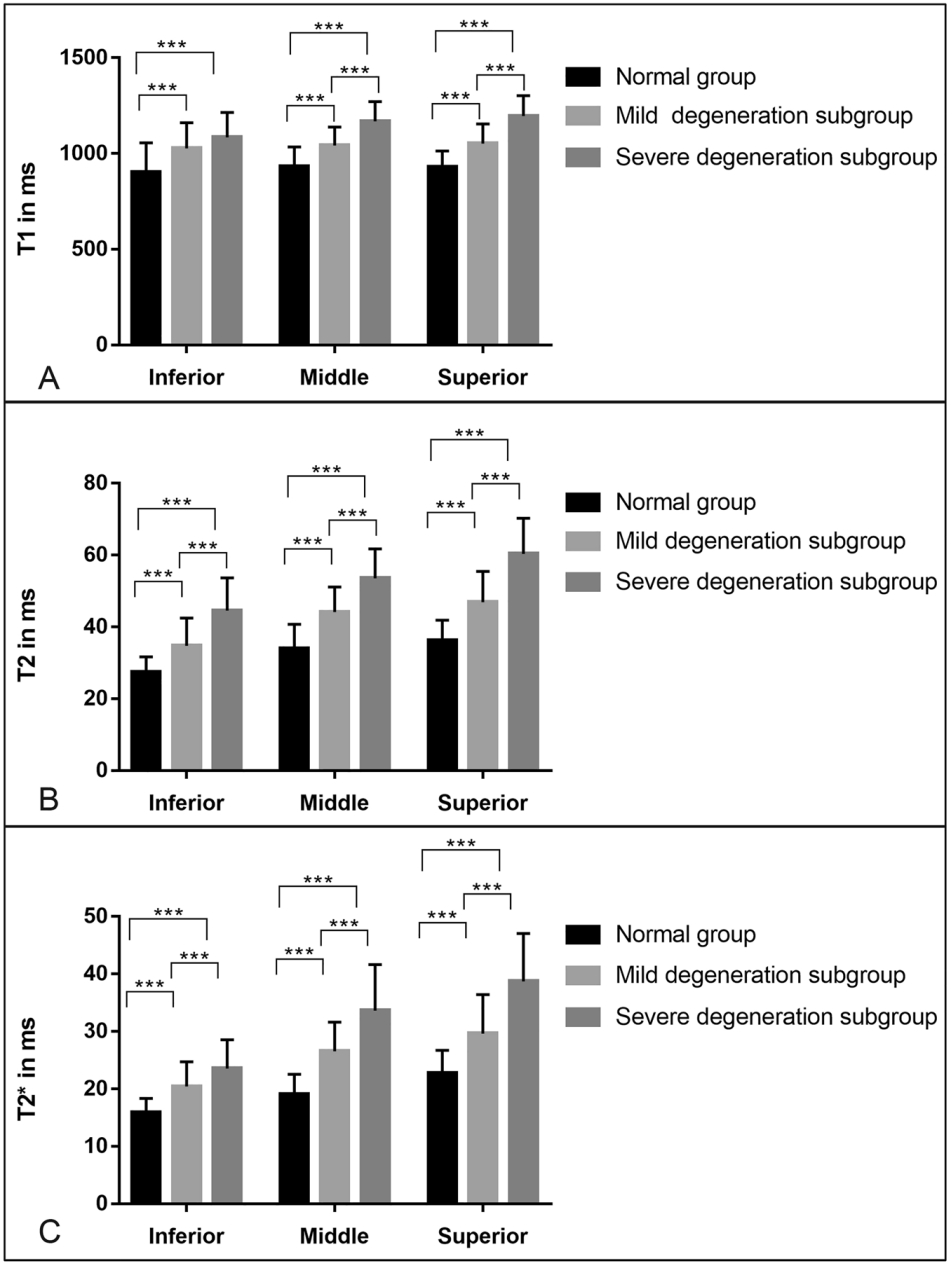
Bar diagram demonstrating average T1, T2, and T2* relaxation times in normalgroup,mild and severe degeneration subgroups. ***P < 0.001.

T1, T2 and T2* mapping can be sensitive to identifying shoulder cartilage degeneration (all *P* < 0.01). The T1, T2 and T2* relaxation times of the superior, middle and inferior bands in the patient group (mild and severe degeneration subgroups) were higher than those in the normal group.No significant difference was observed in the T1 relaxation times of the inferior band of the shoulder joint between the mild and severe degeneration subgroups (*P* = 0.074), but the pairwise comparison of other groups showed a statistically significant difference (*P* < 0.01). The T1, T2 and T2* relaxation times were increased gradually as the destruction and degeneration of the shoulder joint cartilage aggravated, and the difference was statistically significant (Fig. 7).

## Discussion

To our knowledge, Arthroscopy of OA is considered to be the gold standard of osteoarthritis diagnostics^7,8^; however, it is operator-dependent and limited to the evaluation of the articular surface.In order to better evaluate the degeneration of articular cartilage,MRI becomes the only available diagnostic modality for a routine cartilage examination^36^. Krakowski et al^37^ conducted a retrospective analysis of MRI reports and arthroscopic findings was performed on 190 consecutive patients treated in one orthopaedic department,and concluded that the overall diagnostic accuracy in detecting chondral lesions was rather weak, with Kappa values lower than 0.6. In their opinion,MRI image underestimates the extent of cartilage injury and evaluation of cartilage defects. On the other hand, Kohayama et al^35^ reported that agreement between the MRI-based staging system and the ICRS classification was 88.9%, with a sensitivity of 98.4%, specificity of 84.2%, PPV of 95.3%, and NPV of 94.1% for diagnosing an unstable lesion. The MRI-based staging system corresponded well with the ICRS classification. On the contrary, Casula et al^38^'s research showed that qMRI parameters were not linearly related to arthroscopic grading. The severity of cartilage degeneration may not be revealed solely by diagnostic arthroscopy, and thus, qMRI can have a role in the investigation of cartilage degeneration.These quantitative MRI techniques seem to be the most promising due to providing insight into the histologic composition of the cartilage.

In this study, Basing on MRI images, according to the standard ICRS grading, the 123 shoulder cartilages were divided into five grades: Grade 0, Grade 1, Grade 2, Grade 3, and Grade 4. And the T1, T2 and T2* relaxation times of shoulder cartilage were compared between the normal and patient groups, and the importance of quantitative analysis in the degeneration of shoulder cartilage was discussed.There were significant differences in the T1, T2 and T2* relaxation times of the superior, middle and inferior bands of shoulder cartilage in the five grades. With the aggravation of cartilage degeneration, T1 and T2 mapping values showed an increasing trend, which was consistent with previous studies^39,40^. Compared with delayed Gadolinium-enhanced MR cartilage imaging (dGEMRIC), Sodium imaging and other cartilage imaging techniques, T1, T2 and T2* mapping do not require the injection of gadolinium contrast agent, and are highly operable and commonly applied^41,42^. The data of this study showed that 46 shoulder joints in the normal group had MRI-based grade 0, 49 patients (63.6%) had grade 1–2 (mild degeneration group), and 28 patients (36.4%) had grade 3–4 (severe degeneration group), among which the proportion of early mild cartilage degeneration was the highest, which was consistent with previous findings^26^. Bittersohl collected humeral head specimens from 15 patients who underwent shoulder arthroplasty due to osteoarthritis and conducted T2* mapping and dGEMRIC analysis, and the results demonstrated a significant correlation between T2* and T1Gd relaxation times and histological cartilage grade (r-values ranging from 0.315 to 0.784)^26^.

To avoid the influence of water content changes with a load between day and night on T1, T2 and T2* relaxation times, the subjects should undergo the scanning after resting for 30 min at the same time. In this study, T1 relaxation times of the superior, middle and inferior bands of shoulder cartilage were positively correlated with MRI-based grading at a medium–high degree, while T2 and T2* relaxation times were positively correlated with MRI-based grading, which are consistent with the T2* relaxation times of the superior and inferior bands in Bittersohl's study^27^. The T2 relaxation time showed the highest correlation, suggesting that the T2 mapping value has the greatest significance for the quantitative analysis of articular cartilage degeneration, which is consistent with the results of Quatman et al.^36,43–45^ in the evaluation of T2 mapping for early cartilage injury. This study showed that the correlation between T1, T2 and T2* relaxation times of the superior band of shoulder cartilage and MRI-based grading was higher than that of the corresponding middle and inferior bands, and the degeneration of articular cartilage was mainly caused by microscopic changes in the weight-bearing area of articular cartilage. In Casula et al^38^'s research, T1 and T2 relaxation times were found to vary statistically significantly between arthroscopic grades ICRS0–ICRS2, a mild correlation, however, was found when ICRS0 group was excluded from the analysis, which was different from our study. ICRS classification was generally based on arthroscopy or histology ,but MRI-based grading in our study was based on MRI images with ICRS as reference, and did not involve arthroscopy or histology. Contrary to arthroscopy, T1, T2 and T2* mapping visualizes the full thickness of cartilage and provides a quantitative surrogate for alterations in tissue composition and structure, namely disorder and destruction of collagen fiber structure,proteoglycan loss, and increase in water content. Such changes are expected to be asymptomatic at early stages of OA and may also be invisible under arthroscopy. In this study, MRI-based grade of shoulder articular cartilage was positively correlated with age, which was consistent with the rule of degenerationThe T1, T2 and T2* average relaxation times showed a moderately positive correlation with age, which was consistent with Goto's study^46^.

In this study,the T1, T2 and T2* relaxation times in mild and severe degeneration groups were significantly higher than those in normal group, which are consistent with previous studies^22,40^. Previously, cartilage degeneration has been associated with prolonged T1 and T2 relaxation times as compared to normal tissue^47,48^. In the present study,T1, T2 and T2* relaxation times showed a similar increasing trend as the grading increases.It has been reported that increased water content is one of the first changes when articular cartilage deforms. The articular cartilage's T1 and T2 relaxation times are mainly affected by water content in cartilage^18^. The accurate measurement of water content by T1 mapping may be helpful to accurately evaluate the edema during cartilage degeneration in clinical practice, which is complementary to T2 mapping and other biochemical imaging methods of articular cartilage. T2 mapping can reflect the water content of articular cartilage and monitor the microstructure of cartilage, namely, the integrity of the collagen network. It has been widely studied for its potential use in the early diagnosis of cartilage degeneration^25,39,49–51^. It is a relatively mature biochemical imaging technology of articular cartilage, and these advantages are not found in T1 mapping. T2* mapping is more sensitive to the collagen structure of cartilage rather than water content. The imaging time of T2* mapping is shorter than that of T2 mapping, which is more conducive to clinical applications. T1, T2 and T2* mapping can quantitatively analyze the changes in the proteoglycan, water content and collagen structure of cartilage, respectively. With the aggravation of cartilage degeneration, the levels of collagen and proteoglycan decreased, while the water content increased, and the abnormal structure of collagen was detected. The three methods can indirectly reflect the changes in tissue chemical components of shoulder cartilage degeneration from different aspects. Therefore, the combination of the three diagnostic methods can improve the diagnostic accuracy of shoulder cartilage injury.

Shiguetomi-Medina et al.^17,18^ proved that the correlation between water content in the endochondral and T1 value can be used to evaluate the water content in cartilage. The T1 value of articular cartilage is mainly affected by water content and proteoglycan content^19^. These theories provided the basis for the assessment of early articular cartilage injury by using T1 mapping.In this study, we found that the T1 relaxation time of the superior, middle and inferior bands in the patient group (mild and severe degeneration subgroups) were higher than those in the normal group. With the aggravation of the degree of degeneration, T1 relaxation time has an upward trend.This suggests that the change of matrix composition in articular cartilage at early stage can affect T1 relaxation time without Gd-DTPA injection. The change of arrangement pattern in collagen fibre caused an increase in water molecule content in cartilage extracellular matrix (ECM). Furthermore, mucin loss is associated with the inhibited synthesis of cartilage matrix. The less GAG and more water molecules in the medium, the higher the corresponding T1 value. Therefore, the T1 relaxation time can be measured by T1 mapping to quantify the degeneration of shoulder cartilage. At present, there are few studies on T1 mapping without Gd-DTPA injection, and recent research on T1 mapping technology is more concentrated on Gd-DTPA imaging technology^52–55^. Gd-DTPA imaging technology can be used to reflect the changes in T1 relaxation time affected by GAG content, but T1 mapping technique with Gd-DTPA injection costs higher than that without Gd-DTPA injection. Before examination, it requires long preparation time, and more notably, Gd-DTPA has certain side effects, such as renal system fibrosis (NSF), etc. However, the increase of water content in articular cartilage degeneration is small, which may reduce the accuracy of T1 mapping in diagnosing early cartilage degeneration. In a pairwise comparison after the event, the difference in the T1 relaxation time of the inferior band between the mild and severe degeneration subgroups was not statistically significant (*P* = 0.074). We speculate that the influence of pressure on cartilage water content also interferes with the diagnosis of cartilage degeneration by T1 mapping .In addition, inferior band is a non-load-bearing area, so the T1 value may not able to distinguish the degree between the mild and severe degeneration subgroups.

Limitations: there is certain subjective judgment in diagnosing cartilage degeneration based on the MRI imaging. No arthroscopy is available as the gold standard control, which has certain limitations in diagnosing shoulder cartilage degeneration. At the same time, the selection of research objects and areas of interest is subjective to a certain extent. There are fewer males and more females in each group, and T1, T2 and T2* mapping values are mainly analyzed instead of different genders and sequences. Due to limited technical conditions, T1 Gd-DTPA technology and Tlp mapping technology could not be used for imaging.In the future work, we will continue to carry out relevant research, expand the research methods and contents, and make this research work more perfect.

In conclusion, unlike conventional MR image, T1, T2 and T2* mapping image not only provide visual observation of the morphology of articular cartilage and the signal, but also quantitatively reflect the levels of different biochemical components in articular cartilage. Quantitative T1, T2, and T2* mapping sequences can be used as an important supplementary sequence for MRI images of shoulder cartilage,and provide effective guidance to early clinical diagnosis and treatment of shoulder cartilage degeneration.

## Supplementary Information

Supplementary Information.

## Data Availability

The datasets used and/or analysed during the current study available from the corresponding author on reasonable request.
