# Supplementary material for: Application of quantitative T1, T2 and T2* mapping magnetic resonance imaging in cartilage degeneration of the shoulder joint
Source: Sci Rep. 2023 Mar 20;13:4558. doi: 10.1038/s41598-023-31644-2 (PMC10027866; doi:10.1038/s41598-023-31644-2)
Supplement: Supplementary file 1 — Supplementary Information. [file 41598_2023_31644_MOESM1_ESM.docx]

**Supplemental Table 1.** ICRS grading of shoulder cartilage

| **Grade** | **Cartilaginous manifestations** |
| --- | --- |
| Grade 0  Grade 1  Grade 2  Grade 3  Grade 4 | Smooth cartilage surface, no defects, intrachondral signal is uniform, no abnormal subchondral bone signal  Smooth cartilage surface, no defects, a local abnormal signal in subchondral bone  The localized defect on the cartilage surface, not reaching 50% of the whole cartilage  Cartilage defect, more than 50% deep to the whole layer  Total cartilage loss, exfoliation, and exposure of subchondral bone |
